# Supplementary material for: Lupenone, a wonder chemical obtained from Euphorbia segetalis to boost affinity for the transcriptional factor escalating drought-tolerance in Solanum Lycopersicum: A cutting-edge computational biology approach
Source: PLoS One. 2023 Nov 8;18(11):e0281293. doi: 10.1371/journal.pone.0281293 (PMC10631687; doi:10.1371/journal.pone.0281293)
Supplement: Attachment — Submitted filename: Response to Reviewers[16 jan].docx [file pone.0281293.s001.docx]

**PONE-D-22-34473**

Lupenone, a wonder chemical obtained from Euphorbia segetalis to boost affinity for the transcriptional factor escalating drought-tolerance in Solanum lycopersicum: a cutting-edge computational biology approach

PLOS ONE

Thank you very much for your letter with reviewer comments. We are pleased to know that our manuscript was rated as potentially admissible for publication in the PLoS one*,* subject to adequate revision according to reviewer instructions.

We have completed all corrections and revised the manuscript based on the comments made by the reviewers point by point. So, I am sending herewith our revised manuscript with related documents in your favor. We would like to thank you for allowing us to resubmit a revised version of the manuscript. Please inform if any inconvenience or any other suggestions from your end.

I would be highly glad if you take the necessary actions regarding our revised manuscript to publish in your PLoS one.

**Authors Responses to Questions**

**Comments to the Author**
Reviewer #1: The manuscript is very novel and sounds very interesting, few comments must be answered for its better enhancement:
1. Authors must take care of typo errors and grammar errors.

**Response**: Thank you for the progressive comment, we have corrected all typos and grammar errors.
2. Authors should add MMGBSA in their study.

**Response**: Thank you for the progressive comment, we have added MMGBSA in our revised version.
3. Authors must add few more details of drought and its impact.

Response: Thank you for the progressive comment, we have added some more information about it.

Reviewer #2: The manuscript seems interesting and paves a new approach to plant drought tolerance, but needs some modifications as follows:
1. The abstract must be concise;
**Response**: Thank you for the progressive comment, we have made it crisp.
3. Is there any possibility of commercializing Lupenone???

**Response**: Yes, it can easily commercialize.
4. Make the introduction more crisp which may reflect the cause and the new invention in a flowchart manner;

**Response**: Thank you for the progressive comment, we have made it crisp.
5. Increase the DPI of RMSDs;

**Response**: We have increased the DPI’s
6. Add the data of the Free energy landscape;

**Response**: Thank you for the progressive comment, we have added it in revised version.
7. Add binding free energy calculation by MMGBSA/MMPBSA

**Response**: Thank you for the progressive comment, we have added MMGBSA in our revised version.
